# Computer literacy and attitudes towards e-learning among first year medical students

**DOI:** 10.1186/1472-6920-6-34

**Published:** 2006-06-19

**Authors:** Thomas Michael Link, Richard Marz

**Affiliations:** 1 Core Unit for Medical Education, Medical University of Vienna, Vienna, Austria

## Abstract

**Background:**

At the Medical University of Vienna, most information for students is available only online. In 2005, an e-learning project was initiated and there are plans to introduce a learning management system. In this study, we estimate the level of students' computer skills, the number of students having difficulty with e-learning, and the number of students opposed to e-learning.

**Methods:**

The study was conducted in an introductory course on computer-based and web-based training (CBT/WBT). Students were asked to fill out a questionnaire online that covered a wide range of relevant attitudes and experiences.

**Results:**

While the great majority of students possess sufficient computer skills and acknowledge the advantages of interactive and multimedia-enhanced learning material, a small percentage lacks basic computer skills and/or is very skeptical about e-learning. There is also a consistently significant albeit weak gender difference in available computer infrastructure and Internet access. As for student attitudes toward e-learning, we found that age, computer use, and previous exposure to computers are more important than gender. A sizable number of students, 12% of the total, make little or no use of existing e-learning offerings.

**Conclusion:**

Many students would benefit from a basic introduction to computers and to the relevant computer-based resources of the university. Given to the wide range of computer skills among students, a single computer course for all students would not be useful nor would it be accepted. Special measures should be taken to prevent students who lack computer skills from being disadvantaged or from developing computer-hostile attitudes.

## Background

Computer literacy has been a subject of educational research ever since personal computers were introduced to the classroom, either as teaching aids or as tools for self-study. In the 1980s, research on computer literacy focused on the question whether medical students were ready for the foreseeable omnipresence of computers in the future doctors' professional environments [1-4], i.e., whether they possessed the necessary computer skills [2,5-9]. The vision of a knowledge-based society saw future economic wealth dependent on people's abilities to deal with the growing information load and to adapt to an ever-changing working environment [10-13]. It was assumed that computers would become ubiquitous tools for managing medical knowledge [14]. In some medical schools, a privately owned computer was made a requirement for medical students [15,16].

E-Learning, in particular the use of learning management systems (LMSs), introduced a new aspect. Researchers [17] suggested that some students may lack the necessary skills to use web-based learning platforms effectively and are therefore handicapped. This issue is often discussed in the context of gender differences. The main concern is that female students are at a disadvantage due to different patterns of computer usage, e.g. a less dominant style of discussion in web-based communication [18,19]. These gender differences can be observed in students' computer-related behaviors but also in their attitudes towards computer-based and web-based training (CBT/WBT). In a Danish study, Dørup [9] reported that among first-year students, 46% of the men were in favor of replacing "traditional teaching with use of computers if possible" while only 22% women agreed with this statement.

In 2004, 80% of Austria's 20–29 year olds had Internet access and 75% of university and high school students used a computer daily [20]. We can thus assume that, in general, students entering university have good basic computer skills. Studies nevertheless demonstrate that there is a considerable difference in computer use according to students' disciplines. Middendorff [21] reports that German medical students spend an average of 8 hours per week at the computer (including private activities). This is the lowest value of all disciplines, what makes it difficult to draw conclusions about medical students' computer use from general surveys. Often the degree of "informational fluency" remains at a basic level and students tend to over-estimate their computer skills [22].

This study examines the level of computer literacy and patterns of computer usage of first-year medical students at the Medical University of Vienna. It was conducted in an introductory course for first-year students on CBT/WBT. The goal of the study was to determine the need for such introductory courses and to provide information that could be used to improve them. A secondary aim was to identify difficulties that may be encountered in implementing a university-wide LMS due to students' lack of computer literacy or low acceptance of e-learning. While multimedia learning programs have been praised for their educational superiority, actual use of these programs has sometimes failed to meet our expectations.

## Methods

### Subjects

Since autumn 2003, we have required students to take an introductory course on CBT/WBT as a single 90-minute class session. This course is held for first-year students (about 1500 students took it in 2004 and 2005) and second-year students (about 600 students from 2003 to 2005) [23]. The course serves two main purposes:

(1) To ensure a certain level of computer and information literacy, including online communication skills.

(2) To acquaint students with computer and web-based learning materials.

In 2003 and 2004, students had to review web-based learning programs (e.g. [24]) and post their statements in a dedicated online forum. In the course for first-year students we used a student-developed platform [25]. In the course for second-year students, we used Manila [26] in 2003 and TikiWiki [27] in 2004 as a collaboration tool. In 2005, we switched to tools that were partly self-developed and less demanding with respect to the server load.

This paper reports on data from an online survey for the 2004 course for first-year students. Participation in the survey was voluntary and anonymous (though students were asked to give their student ID if they wanted to). The tutors were not able to determine who has or has not filled out the questionnaire. Using class time for students to fill out the questionnaire nevertheless ensured a high response rate of 79%.

A total of 1232 questionnaires were completed, 1160 of which remained in the data set after applying some filtering rules in order to eliminate records of uncertain origin. The gender breakdown of respondents was 61% female and 39% male. This corresponds exactly to the gender breakdown of the 1560 students entering the study module (61% female and 39% male). We thus conclude that our sample was representative of the 2004 cohort. Missing values due to non-responses are not included in tables or figures. Differences between the reported counts and the sample size (n = 1160) are thus due to missing responses.

### Questionnaire

The questionnaire [28] (see Additional file 1) was designed to collect the following information:

(a) Overall evaluation of the course

(b) Attitudes towards e-learning as well as previous experiences and expectations about the use of CBT/WBT

(c) Computer and Internet usage

(d) Extent of students' private computer infrastructure

(e) Basic demographic data.

In the following, we will focus on students' computer usage and private computer infrastructure as well as their attitudes toward e-learning.

Attitudes towards e-learning (understood as an umbrella concept for learning methods supported by information- and communication technologies (ICT) in general) were determined by the students' agreement or disagreement with several statements about the importance of ICT in medical education. These statements contained items like "Web-based learning programs are able to replace lectures" or "In medical teaching, there is no need for the use of Web-based programs." The students rated their agreement or disagreement on a bi-polar eight-point Likert scale. For the purpose of comparability with Dørup [9], we recoded their answers into dichotomous variables. As computer use and attitudes towards e-learning were measured on an ordinal scale, we accordingly used Spearman rho to describe the statistical relationship of these variables with other items. For other metric variables Pearson r was used.

## Results

### Computer infrastructure

Almost all students (94%) have access to a privately owned PC they can use for their studies, which is either owned by the students themselves (74%) or shared with family members or roommates (20%). Only 5% rely primarily on public computer facilities (Table 1).

Student-owned PCs are on average 2.3 years old; 92% are newer than 5 years, 87% newer than 4 years. This corresponds to the life span of computers in companies or public administration offices. Only 3.2% of the students have a computer older than 6 years. Male students' PCs (mean ± SD: 2 ± 1.42 years) are newer than those owned by women (2.5 ± 2.05 years). The 95% confidence interval for the difference is 0.33..0.79 years.

### Internet access

The great majority of students also have access to the Internet, though the quality of connectivity varies widely; 60% have access via ADSL, cable TV, or LAN (which, however, usually signifies the use of public facilities at the university or elsewhere); 37% have access using a telephone connection (modem or ISDN) (Table 2). The type of Internet access differs according to gender (Cramer V = 0.28, p = 0.001). Male students tend to have faster Internet access while older technologies (e.g. modem) are more common among women. The proportion of modem users is twice as high among women (33%) than among men (15%).

### Computer use

#### Types of computer use

Students are familiar with e-mail and the use of the Internet for information research; 94% of the students communicate via e-mail and 97% use the Internet for information research at least several times per month. While the use of word processors is very common (82% use such a program several times a month), students are less familiar with other program types (Table 3).

Very few medical students have experience in Web design or the creation of HTML documents (5% at least weekly) and thus make no use of the Internet for publishing or more sophisticated collaboration purposes. The frequencies of using communication technologies other than e-mail, e.g., chats (21%), forums and bulletin boards (13%), are also low.

One noteworthy detail is the proportion of students who use computers for organizing appointments, to do lists, or making notes: 28% use such a personal organizer software several times per week, which may point to the use of personal digital assistants (PDA) or smart cell phones.

Except for the categories "Word Processor" and "E-mail," male students use the computer significantly more often than women. The strength of this statistical relationship is weak. Spearman rho is highest for the categories "Web-design" (r_s _= 0.25, p = 0.001), "Games" (r_s _= 0.23, p = 0.001), "Forums" (r_s _= 0.21, p = 0.001), and "Spreadsheets" (r_s _= 0.20, p = 0.001).

#### Age when using a computer for the first time

Half of all students (50%) used a computer for the first time by the age of 11 (mean 11.2 ± 3.77 SD). By the time they entered university, i.e., before the age of 18, fully 96% of all students had begun to use computers. The average age when students began using computers for the first time is slightly lower for men (10.7 ± 3.40 years) than women (11.5 ± 3.96 years). The 95% confidence interval for this difference is 0.33..1.24 years.

### E-Learning

#### Prior experiences and expectations

Half of the students (49%) report using a computer or Web-based learning program at least once per month. In order to determine how many students have little or no experience with e-learning, we consolidated answers to questions about four different kinds of e-learning programs (information retrieval, downloading scripts, LMS, and CBT/WBT) into one index. Because of the high response rates for "downloading learning material," we defined inexperienced users as those who answered "less often" or "never" to questions about at least three of these kinds of programs. Following this typology, 12% of the students are inexperienced, having used at most one kind of e-learning program at least once per term (Table 4).

The majority of students (66%) have already used a computer or Web-based dictionary like the Pschyrembel medical dictionary, which is one of the standard references used by Vienna medical students. Half of them (50%) have used an online image repository at least once and 42% have used some kind of online quiz to test their knowledge (Table 5). Other kinds of learning programs, such as those associated with a constructivist approach, are less well known among first-year Vienna students. The results given in Tables 4 and 5 relating to students' use of LMS are inconsistent. This inconsistency arises most likely from the students' lack of understanding of what a LMS is since very few lecturers use this kind of software to support their courses.

About 10% of the students have never used any of the above-mentioned kinds of e-learning programs and 4.4% do not regard any of them as helpful. Those who regard only two or fewer as helpful tend to prefer learning programs that have no "built-in" educational theory, such as encyclopedias (38%), image collections (23%), and quizzes (23%). The number of different kinds of programs that students have experience with and that they consider helpful correlates with Pearson r = 0.32 (p = 0.001) – the more kinds of programs they know, the more kinds they consider useful.

#### Attitudes

**Figure 1:**
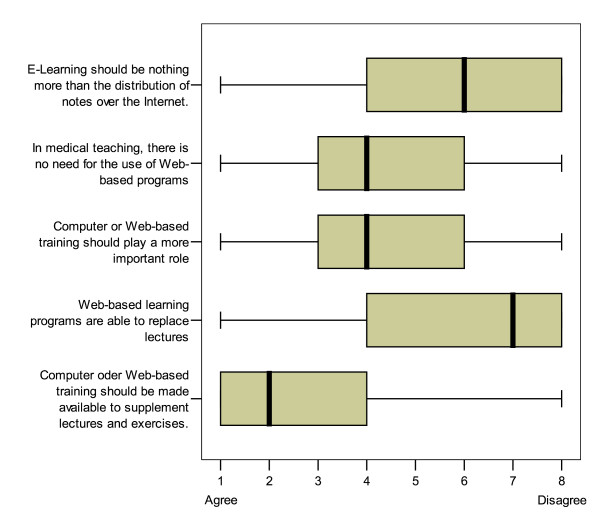
**Students' agreement or disagreement with statements on the usefulness of e-learning**. The x-axis represents the values of an 8-point bi-polar rating scale: 1 = strong agreement, 8 = strong disagreement. The boxes show the quartiles (25% of the distribution) and the median (50% cut).

A majority of the students agree (median = 2, interquartile range = 3) that CBT/WBT should be offered as a supplement to lectures and seminars (Figure 1). On the other hand, most students disagree with the statement that e-learning should replace these traditional forms of teaching (median = 7, IQR = 4).

Men (median = 6) tend to be slightly more in favor of replacing traditional lectures with CBT/WBT than women (median = 7). The strength of this effect is negligible (r_s _= 0.06, p = 0.041). After recoding to a dichotomous scale (1..4 = pro, 5..8 = contra), 28% of male and 25% of female students can be considered favoring the replacement of traditional teaching methods with e-learning. The gender difference is slightly bigger for the item "Computer or Web-based training should play a more important role" but still hardly noteworthy (r_s _= 0.16, p = 0.001). In general, the following variables have bigger effects on e-learning-related attitudes than gender per se:

(a) Lack of experience with CBT/WBT

(b) Age

(c) Productive computer and Internet use (e.g. spreadsheets, organizer, word processor, graphics, e-mail, Web design, and information research).

We consolidated statements 2 to 4 in Figure 1 into one index (Cronbach alpha = 0.65; inclusion of the items 1 and 5 leads to a slight decrease in reliability). In a regression model (Table 6) that includes the above 3 variables and gender (R^2 ^adj = 0.15, p = 0.001, SEE = 1.54), gender is not statistically significant (p = 0.41). When the stepwise regression method is used, gender is excluded from the final model.

## Discussion

### Computer infrastructure and internet access

A sizable number of students still have Internet access only via dial-up connections using a modem. This mode of Internet access is slow and impedes the use of synchronous communication tools that require one to stay online for a long period of time. Even if the majority of students do have broadband access to the Internet, mandatory e-learning solutions cannot rely on synchronous online communication tools like chats and on extensive video material, e.g. recordings from lectures. Instead, preference should be given to asynchronous online communication tools and textual information along with videos. Asynchronous communication tools also have the advantage that teachers and students do not have to be online at the same time.

### Computer use patterns

Only a small number of students have experience with Internet publishing and asynchronous communication tools like BBS or forums. Thus, most of our students are rather passive Internet users and miss out on numerous possibilities of virtual communities and Web-based publishing. The lack of experience with synchronous and asynchronous online communication, with the exception of e-mail, may cause problems when using the collaboration tools included in an LMS [29].

### Attitudes towards e-learning

Most students agree that e-learning could serve as a supplement for lectures and seminars. However, about as many students disagree with the statement that e-learning could replace traditional ways of teaching. In the Danish context, Dørup [9] reported a slightly greater proportion of first-year medical students in favor of replacing traditional lectures with e-learning (47% men, 22% women). These higher levels of agreement could be explained by the different response scales used but also by the fact that Danish people in general are reported [30] to be more "digital literate" than Austrians – although this difference cannot be claimed for persons under 24 years of age [30].

The intensity of computer use and previous experience with CBT/WBT have the greatest effect on students' attitudes towards e-learning. The explanation for this could be a general discomfort with the technology that makes students who lack experience with ICT express themselves cautiously about its use in education [31]. It could also be explained by the relative novelty of e-learning and students' difficulties in integrating CBT/WBT into their way of learning [32].

Most students seem to acknowledge the range of possibilities of new media to enhance their learning experience although they consider CBT/WBT a supplement to rather than a replacement of other learning materials. However, there is also a group of students who are strictly opposed to CBT/WBT (4.4% of the first-year students do not value any of the kinds of programs mentioned above). More disturbing, 24% strongly agree (values 1 and 2 on an 8-point rating scale) with the statement that the Medical University of Vienna could do well without CBT/WBT. When introducing an online LMS or Web-based learning program, special care should be taken not to lose these students because of the choice of a certain learning technology.

In December 2005, we also held a few focus groups with teachers and students on a similar subject. In the course of these discussions it became clear how some characteristics of the new curriculum, especially the emphasis on the MCQ-based year-end examinations, impeded the use of CBT/WBT. In these discussions the students had doubts about the usability and efficiency of e-learning (with regard to costs, handling of ICT, but also learning efficiency) while they still acknowledged the possibilities of ICT support with respect to visualization, simulation, self-quizzing, and fast information retrieval from several sources such as encyclopedias or Web pages.

### Gender differences

We were able to identify gender differences for all computer-related variables. In sum, men make more frequent use of computers and have access to better computer infrastructure and faster Internet connections. While this difference is quite consistent over several variables, the strength of the statistical relationship is weak and, with respect to students' attitudes towards e-learning, overshadowed by other variables (e.g. previous exposure to CBT/WBT) that are more important for predicting students' attitudes.

With respect to the implementation of an LMS, the most important difference between men and women is the relatively high number of women still using a slow dial-up connection to the Internet, which could impede the use of synchronous communication tools or multimedia-rich Web applications. Well planned use of e-learning and supportive measures should help to neutralize this difference. Although women have less experience with forums, Gunn [19] showed that these differences in online communication behavior do not necessarily result in worse examination outcomes.

## Conclusion

E-Learning must be appropriate to students' level of computer expertise in order not to become a source of frustration. Courses to develop students' computer skills can improve this situation by influencing students' attitudes and capabilities. Our conclusions with respect to such introductory courses are twofold. Students certainly need some kind of formal introduction to the new ICT for learning purposes. But due to the wide range of previous experience and computer skills, there is no one-size-fits-all course design available. Such a course should either be split into several tracks according to students' different levels of computer literacy [33], or it should be held only for students with little or no computer experience.

There is, however, the danger that precisely those students who need this course the most will hesitate to attend it voluntarily. It is difficult to say how these students could be persuaded to take such a course despite their skepticism towards ICT and e-learning. One strategy would be to emphasize the practical value for solving everyday problems and obtaining useful information. Once they have learned how computers help them solve recurring problems, they will perhaps develop more computer-friendly attitudes. Another solution could be to make the course compulsory but to make the impact negligible for students with good ICT knowledge. This could be achieved with a Web-based entry test. Students who pass the test would be exempted from having to take the course.

When introducing a campus-wide LMS, one has to take into consideration that some students lack the necessary computer skills or infrastructure to participate effectively in online courses, and that others are strictly opposed to e-learning. Introducing a campus-wide e-learning solution thus poses not only technical and organizational challenges but also calls for a promotional strategy. In the future, we can expect more students to think of computers as standard tools for learning as schools make more use ICT in their classrooms. For example, an "avant-garde" of Vienna medical students already created an online forum [34-36] for informally exchanging information about courses as well as students authored learning materials.

## Competing interests

The author(s) declare that they have no competing interests.

## Authors' contributions

RM and TML planned and organized courses [23] to promote computer literacy among medical students.

TML was responsible for designing the study, implementing the online questionnaire, analyzing the data, writing the first draft, and proofreading the final draft.

RM was responsible for designing the course content, recruiting and training the tutors and supervising all aspects of the course. He revised the article extensively.

Both authors read and approved the final version.

## Pre-publication history

The pre-publication history for this paper can be accessed here:



## Supplementary Material

Additional File 1 This is the HTML output of the English translation of the questionnaire we used. The HTML was originally generated by a PHP script. As this is only the HTML representation of the questionnaire, clicking on the "Submit" button has no effect. The file can be viewed in any Internet browser. Click here for file

## Figures and Tables

**Table 1:** Computer infrastructure available to first-year Vienna medical students

	No. (%) of students	
Own Personal Computer	851	(73.9)
Family, student apartment	233	(20.2)
Public computer room	59	(5.1)
None	9	(0.8)
Total	1152	(100.0)

**Table 2:** Type of Internet access for first-year medical students

	Men		Women		Total	
	No. of students (%)		No. of students (%)		No. of students (%)	

Modem	67	(15.1)	228	(32.8)	295	(25.9)
ISDN (Integrated Services Digital Network)	37	(8.3)	87	(12.5)	124	(10.9)
Cable, ADSL (Asymmetric Digital Subscriber Line), etc.	291	(65.5)	264	(37.9)	555	(48.7)
LAN (Local Area Network)	39	(8.8)	93	(13.4)	132	(11.6)
None	10	(2.3)	20	(2.9)	30	(2.6)
Not applicable	0	(0.0)	4	(0.6)	4	(0.4)
Total	444		696		1140	

**Table 3:** Computer and Internet use among first-year medical students

	Daily		Several times a week		Several times a month		Less often		Never		Total
	No. of students (%)		No. of students (%)		No. of students (%)		No. of students (%)		No. of students (%)		No. of students

Word Processor	267	(23.3)	392	(34.2)	287	(25.0)	187	(16.3)	15	(1.3)	1148
Organizer	180	(15.9)	133	(11.8)	92	(8.1)	319	(28.2)	406	(35.9)	1130
Games	74	(6.5)	125	(11.0)	177	(15.6)	412	(36.3)	348	(30.6)	1136
Draw, paint	64	(5.6)	194	(17.0)	351	(30.8)	349	(30.6)	183	(16.0)	1141
Spreadsheets	35	(3.1)	50	(4.5%)	141	(12.6)	412	(36.7)	485	(43.2)	1123
E-mail	509	(44.4)	433	(37.8)	140	(12.2)	56	(4.9)	9	(0.8)	1147
Information	480	(42.0)	484	(42.3)	150	(13.1)	27	(2.4)	2	(0.2)	1143
Chat	121	(10.7)	118	(10.4)	77	(6.8)	305	(26.9)	515	(45.3)	1136
BBS (Bulletin Board Systems), Forum	69	(6.1)	83	(7.4)	117	(10.4)	379	(33.5)	482	(42.7)	1130
Web design	21	(1.9)	34	(3.0)	42	(3.7)	264	(23.4)	768	(68.0)	1129

**Table 4:** Frequency of e-learning use among first-year medical students

	At least weekly		At least monthly		Once per term		Less often		Never		Total
	No. of students (%)		No. of students (%)		No. of students (%)		No. of students (%)		No. of students (%)		No. of students

Downloading scripts	694	(61)	360	(31)	33	(3)	34	(3)	24	(2)	1145
Information research	625	(54)	378	(33)	34	(3)	88	(8)	24	(2)	1149
Computer- or web-based learning programs	199	(17)	356	(31)	136	(12)	302	(26)	148	(13)	1141
Learning-management systems	139	(12)	270	(24)	138	(12)	327	(29)	260	(23)	1134

**Table 5:** Number (%) of students having experiences with certain kinds of learning programs or consider them useful

	Experiences		Considered helpful	
	No. of students (%)		No. of students (%)	

Animations	361	(31.1)	501	(43.2)
Image collections	582	(50.2)	518	(44.7)
Forums	251	(21.6)	251	(21.6)
Hypertexts (e.g. web-based text books)	279	(24.1)	306	(26.4)
Encyclopedias (e.g. Pschyrembel)	767	(66.1)	641	(55.3)
Learning management systems	118	(10.2)	192	(16.6)
Quiz	486	(41.9)	506	(43.6)
Simulations (e.g. patient simulations)	159	(13.7)	402	(34.7)

**Table 6:** Coefficients for a regression model to explain attitudes towards e-learning

	Non-standardized		T	Significance
	B	Standard error		

(Constant)	-1.48	0.38	-3.94	0.00
Gender	-0.08	0.10	-0.83	0.41
Age	0.04	0.01	2.88	0.00
Productive ICT use	0.47	0.08	5.99	0.00
Previous experiences	-0.41	0.05	-8.06	0.00
