# Supplementary material for: Computer literacy and attitudes towards e-learning among first year medical students
Source: BMC Med Educ. 2006 Jun 19;6:34. doi: 10.1186/1472-6920-6-34 (PMC1534040; doi:10.1186/1472-6920-6-34)
Supplement: Additional File 1 — This is the HTML output of the English translation of the questionnaire we used. The HTML was originally generated by a PHP script. As this is only the HTML representation of the questionnaire, clicking on the "Submit" button has no effect. The file can be viewed in any Internet browser. [file 1472-6920-6-34-S1.htm]

Evaluation questionnaire "Computer-based learning"


# Evaluation questionnaire "Computer-based learning"

This questionnaire covers items about your views on computer-based learning as well as attitudes
in general and your living situation. The goal of this online survey is to get a better idea for whom computer-based
learning is a suitable learning instrument and for whom it isn't.

When you have finished filling out the questionnaire, please press "Submit". Only then will your data
be transmitted to the server.

Any information you supply for this survey is of course voluntary.
Since your information helps us in improving our planning, we ask you to take the time to fill out the
questionnaire completely and with care.

The course

Which session did you attend?

|  |  |
| --- | --- |
| Day: | [Please select] Tuesday, 16.11. Wednesday, 17.11. Thursday, 18.11. Friday, 19.11.  Monday, 22.11. Tuesday, 23.11. Wednesday, 24.11. Friday, 10.12. Not applicable (I didn't attend the course) |

|  |  |
| --- | --- |
| Time: | [Please select] 10:15-11:45 12:00-13:30 13:00-14:30 13:45-15:15 14:45-16:15 16:30-18:00 Not applicable (I didn't attend the course) |

To which degree do you think the following items were well or poorly done in this course?

|  |  |
| --- | --- |
| Get a general overview of the possibitlies of computer- and web-based learning materials | Well done         Poorly done |

|  |  |
| --- | --- |
| Get to know a few examples of web-based learning programs | Well done         Poorly done |

|  |  |
| --- | --- |
| Learn to evaluate learning materials on the Internet | Well done         Poorly done |

|  |  |
| --- | --- |
| Learn how to write and publish an evaluation of a web-based learning resource | Well done         Poorly done |

Do you intend to use eMed after this course is over?

Yes  
No

Computer- and web-based learning

What do you think about the following statements? How far do you agree or disagree?

|  |  |
| --- | --- |
| Computer or Web-based training should play a more important role. | I agree         I disagree |

|  |  |
| --- | --- |
| Web-based learning programs are able to replace lectures. | I agree         I disagree |

|  |  |
| --- | --- |
| In medical teaching, there is no need for the use of Web-based programs. | I agree         I disagree |

|  |  |
| --- | --- |
| Computer oder Web-based training should be made available to supplement lectures and exercises. | I agree         I disagree |

|  |  |
| --- | --- |
| I have difficulties or I just don't like working with learning programs which are in English. | I agree         I disagree |

|  |  |
| --- | --- |
| E-Learning should be nothing more than the distribution of notes over the Internet. | I agree         I disagree |

|  |  |
| --- | --- |
| I find it awkward to speak out in the classroom, which is why I often refrain from doing so. I would find it easier to participate in a discussion in an online-forum. | I agree         I disagree |

Different types of learning programs exist. With which of these have you already worked (before
this course)?

Image repositories (usually containing little explanatory
text)  
Hypertexts (e.g. web-based textbooks)  
Simulations (e.g. patient or laboratory simulations)  
Quizzes (e.g. question repositories with assessment)  
Animations (e.g. computer animations which offer some user interaction)  
Encyclopedias (e.g. Online-Pschyrembel)  
Forums for communicating with other students  
Learning management systems (portals for hosting web-based courses)

Other:

Which of these types do you consider the most useful for learning?

Image repositories (usually containing little explanatory
text)  
Hypertexts (e.g. web-based textbooks)  
Simulations (e.g. patient or laboratory simulations)  
Quizzes (e.g. question repositories with assessment)  
Animations (e.g. computer animations which offer some user interaction)  
Encyclopedias (e.g. Online-Pschyrembel)  
Forums for communicating with other students  
Learning management systems (portals for hosting web-based courses)

Other:

Computer use

At which age did you use a computer for the first time (PC, Mac or something similar)?

How often do you use a computer for the following tasks?

|  |  |  |  |  |  |  |
| --- | --- | --- | --- | --- | --- | --- |
| Write texts | |  |  |  |  |  | | --- | --- | --- | --- | --- | | daily | several times a week | several times a month | less often | never | |

|  |  |  |  |  |  |  |
| --- | --- | --- | --- | --- | --- | --- |
| Organize appointments, tasks, and notes | |  |  |  |  |  | | --- | --- | --- | --- | --- | | daily | several times a week | several times a month | less often | never | |

|  |  |  |  |  |  |  |
| --- | --- | --- | --- | --- | --- | --- |
| Create spread sheets or perform calculations | |  |  |  |  |  | | --- | --- | --- | --- | --- | | daily | several times a week | several times a month | less often | never | |

|  |  |  |  |  |  |  |
| --- | --- | --- | --- | --- | --- | --- |
| Create or touch up images (including Fotos) | |  |  |  |  |  | | --- | --- | --- | --- | --- | | daily | several times a week | several times a month | less often | never | |

|  |  |  |  |  |  |  |
| --- | --- | --- | --- | --- | --- | --- |
| Play games | |  |  |  |  |  | | --- | --- | --- | --- | --- | | daily | several times a week | several times a month | less often | never | |

|  |  |  |  |  |  |  |
| --- | --- | --- | --- | --- | --- | --- |
| Send e-mails | |  |  |  |  |  | | --- | --- | --- | --- | --- | | daily | several times a week | several times a month | less often | never | |

|  |  |  |  |  |  |  |
| --- | --- | --- | --- | --- | --- | --- |
| Chat | |  |  |  |  |  | | --- | --- | --- | --- | --- | | daily | several times a week | several times a month | less often | never | |

|  |  |  |  |  |  |  |
| --- | --- | --- | --- | --- | --- | --- |
| Participate in online discussions in forums, BBS, or mailing lists | |  |  |  |  |  | | --- | --- | --- | --- | --- | | daily | several times a week | several times a month | less often | never | |

|  |  |  |  |  |  |  |
| --- | --- | --- | --- | --- | --- | --- |
| Search the Internet for Informationen | |  |  |  |  |  | | --- | --- | --- | --- | --- | | daily | several times a week | several times a month | less often | never | |

|  |  |  |  |  |  |  |
| --- | --- | --- | --- | --- | --- | --- |
| Create a website or publish something on the Internet | |  |  |  |  |  | | --- | --- | --- | --- | --- | | daily | several times a week | several times a month | less often | never | |

|  |  |  |
| --- | --- | --- |
| Other | |  | | --- | |  | |

Computer access

Do you have ready access to a computer you can use *for learning*?

Yes, my own computer  
Yes, a computer shared by a family or in an apartment  
Yes, in a public computer facility (e.g. at the university)  
No

Does this computer have Internet access?

Yes, modem (telephone line)  
Yes, ISDN or similar  
Yes, cable/ADSL or another type of broad-band Internet access  
Yes, LAN (e.g. in public computer rooms at the university)  
No  
Not applicable (e.g. because no computer available)

If you possess a computer of your own, how old (production date) is it?

[Please select]
less than 1 year
1 to 2 years
2 to 3 years
3 to 4 years
4 to 5 years
5 to 6 years
6 to 7 years
7 to 8 years
8 to 9 years
9 to 10 years
10 years and more
Not applicable (e.g. because no computer available)

How often do you use a computer for learning? To ...

|  |  |
| --- | --- |
| ... search the Internet for relevant webpages. | at least weekly at least monthly about once a term less often never |

|  |  |
| --- | --- |
| ... download notes or similar items (with a known Internet address). | at least weekly at least monthly about once a term less often never |

|  |  |
| --- | --- |
| ... use a learning management system for a course (other than the medical university's study guide). | at least weekly at least monthly about once a term less often never |

|  |  |
| --- | --- |
| ... use computer- or web-based learning programs (CD-ROMs, webpages or similar). | at least weekly at least monthly about once a term less often never |

Learning style

The following statements describe diffent kinds of learning strategies.
How far do they apply to your way of learning? Do you rather agree or disagree with
following statements?

|  |  |
| --- | --- |
| I learn by myself most of the time. | I agree         I disagree |

|  |  |
| --- | --- |
| When learning, I usually make summaries and outlines. | I agree         I disagree |

|  |  |
| --- | --- |
| I think of concrete examples to which I can apply the material to be learned. | I agree         I disagree |

|  |  |
| --- | --- |
| I make diagrams and figures to structure the learning material. | I agree         I disagree |

|  |  |
| --- | --- |
| I learn with friends. We explain the subject to each other and quizz each other. | I agree         I disagree |

|  |  |
| --- | --- |
| I try to imagine things as pictures. | I agree         I disagree |

|  |  |
| --- | --- |
| It certainly helps when something explained in a text is illustrated with images and figures. But personally I don't consider such illustrations a necessity to better comprehend the facts. | I agree         I disagree |

Personal information

Are you ...

male  
female

How old are you?

Is German your mother tongue or do you speak German at the level of a mother tongue?

Ja  
Nein

Living

The following two questions are about the town or the city where you
live (your home town) or lived before beginning your studies.

How many inhabitants does this town/city have?

less than 1.000  
1.000 and more  
10.000 and more  
100.000 and more  
1.000.000 and more

How many kilometers is this town/city away from Vienna?

[Please select]
It's Vienna
Less than 10 km
10..19 km
20..29 km
30..39 km
40..49 km
50..59 km
60..69 km
70..79 km
80..89 km
90..99 km
100..199 km
200..399 km
400 km and more

Do you live in Vienna?

Yes, all year long  
Yes, but only during the school-year  
No

Other

Remarks concerning the course, general feedback:

Student ID:

Why do we ask for your student ID? One year from now, we will ask you again to fill
out a questionnaire. Your student ID would enable us to combine these two surveys. The
student ID will be saved in an uni-directionally encrypted form so that one cannot deduce
the actual ID from the saved value. The data will not be given to other people. Your
information will be treated with the greatest condifentiality.

Prompt for confirmation to prevent premature submission of the questionnaire\*

I have filled out the questionnaire and I want to save my information now.

\* denotes required field
